# Three-Dimensional Parametric Analyses of Cross-Passages in Shallow Tunnels Within Noncohesive Soils

**DOI:** 10.1007/s40515-025-00577-w

**Published:** 2025-03-18

**Authors:** Ahsan Saif, Ibtissem Siad, Enrico Soranzo, Wei Wu

**Affiliations:** 1 University of Natural Resources and Life Sciences, Vienna, Austria; 2 University of Sciences and Technology Houari Boumediene, Algiers, Algeria

**Keywords:** 3D finite element, Child tunnel, Cross passage, Hardening soil with small strain, Tunnel intersections, Underground construction

## Abstract

Cross-passages are small-diameter tunnels between running tunnels or between a tunnel and a shaft, essential for safety, maintenance, emergency evacuation, and ventilation. The geotechnical design of these cross-passages is complex and not well understood. This paper presents results from a parametric study carried out in a three-dimensional finite element framework using an advanced nonlinear elasto-plastic constitutive model to represent the surrounding soil. Key parameters varied include the aspect ratio of the cross-passage to the parent tunnel, soil strength, and intersection angle. Findings indicate a significant increase in hoop compression at the springlines and longitudinal tension at the crown and invert of the cross-passage. The aspect ratio significantly affects stress distribution, with maximum stress arching occurring at an aspect ratio of 0.5. With the increase in the opening size, stress redistribution is reduced, and opening deformation is increased. The ideal intersection angle between a cross-passage and parent tunnel is found to be 90°, with angles below 70° leading to a large increase in stress concentrations. Extent of stress redistribution due to cross-passage is found to range from 1.5 to 1.2 times its diameter for large openings in loose soils and smaller openings in dense soils, respectively.

## Introduction

Cross-passages (CPs), often referred to as child tunnels, are common components of modern tunneling, serving as connections between a running tunnel to a shaft or between two running tunnels, known as parent tunnels (PTs). Transport tunnels typically consist of twin tubes, each allowing movement in one direction, interconnected by cross-passages constructed at intervals of 250 to 500 m. These cross-passages serve various purposes including access between tunnels, emergency exits, or housing heavy equipment (ITA-COSUF, 2019). The proper design and construction of cross-passages are crucial for ensuring the safety and integrity of the parent tunnels.

The earliest attempts to quantify stress redistribution around tunnel cross-passages were experimental and involved photoelasticity, undertaken by Riley (1964) and Pant (1971). Subsequently, Brown and Hocking (1976) and Hocking (1978) employed a 3D boundary element (3D-BE) framework to analyze stress concentration factors at tunnel junctions for different tunnel cross-passage geometries. Gercek (1986) consolidated the outcomes of these early investigations and noted that tunnel cross-passages represent structurally vulnerable regions within underground infrastructure and are particularly prone to potential instability. Subsequent investigations have primarily revolved around numerically simulating the impact of cross-passages on the running tunnel. The prevalent approach has been to employ three-dimensional (3D) finite element (FE) and finite difference (FD) analyses, typically in the framework of case-specific studies (Thareja et al. 1980; 1985; Takino et al. 1985).

Tsuchiyama et al. (1988) conducted elastic numerical analyses to evaluate the influence zone around a parent tunnel during the construction of an access tunnel. Pottler (1992) investigated a typical tunnel junction configuration within the English Channel Tunnel Project to determine the necessity for increased support lining thickness near the intersection area. Swoboda et al. (1998) utilized finite element modeling to design and assess the stability of an intersection between the parent tunnel and an escape tunnel in the Schonberg Tunnel Project in Austria. Hsiao et al. (2005) and Sjoberg et al. (2006) employed numerical analyses to examine the behavior of tunnel intersection areas in the Hsuehshan Tunnel in Taiwan and the Citybanan Tunnel in Stockholm, respectively. Particularly, Hsiao et al. (2005) investigated the deformational response of tunnel junctions and recommended reinforcement for specific intersection areas using a combination of numerical analyses and artificial neural networks. Jones (2007) analyzed the distribution of stresses in sprayed concrete shallow tunnel junctions using both in situ monitoring data and advanced numerical modeling.

Similarly, Liu et al. (2009) reported tensile cracking in the shotcrete lining within the zone of influence of the cross-passage opening using 3D analysis. Additionally, Hsiao et al. (2009) conducted 75 3D numerical analyses under various excavation scenarios for intersecting tunnels in rock conditions. They found that the strength/stress ratio of rock significantly impacted stresses near lateral openings in tunnels. Schikora et al. (2013) presented relevant studies demonstrating the potential of optimized design through 3D numerical modeling for tunnel junctions similar to the work of Forder et al. (2008). Kuyt (2014) studied the mechanisms influencing cross-passage behavior based on 3D numerical analysis and monitoring data from the Brisbane Airport link project.

Spyridis and Bergmeister (2015) examined the impact of a perpendicular cross-passage on the structural response of a shallow parent tunnel in a linearly elastic surrounding medium through 3D FE analyses. Their findings indicated that cross-passages induce longitudinal tension in the crown-invert area, compression in the hoop direction on the springlines in the parent tunnel, and stress redistribution extends approximately 1 diameter away from the breakout opening. Moreover, the authors observed that these force-moment concentrations were notably influenced by the surrounding soil stiffness and, to a lesser extent, by the ratio of the parent tunnel and cross-passage diameter. More recent parametric research by Chortis and Kavvadas (2021) utilized 3D FE analyses to explore the impact of cross-passage construction intersecting an existing parent tunnel in a rock mass. The study varied parameters such as the construction sequence of the child tunnel, diameter of the child tunnel (*d*), rock overburden (*H*), in situ horizontal stress ratio (*K*_*O*_), rock mass strength (*σ*_*cm*_), and deformation modulus (*E*_*m*_) to assess their effect on induced axial forces in the tunnel lining near the intersection area.

Cohen et al. (2023) attempted to find a practical solution for determining the construction length of a cross-passage in a 2D finite element analysis and concluded that the excavation length required for 2D analysis is solely dependent on the intersection geometry and is not influenced by the mechanical properties of the surrounding rock. Siad et al. (2023a) found that the umbrella arch technique reduces surface settlements by 30% during cross-passage excavation using 3D FE analyses and data from the Algiers Metro Project. They confirmed the homogenized zone method’s effectiveness for modeling these arches. In another study, Siad et al. (2023b) showed that cross-passage excavation significantly impacts surface settlements and the main tunnel, with larger plastic zones forming around tunnel sidewalls. Thomas et al. (2023) used distributed fiber optic sensor (DFOS) system monitoring system at two junctions at Crossrail’s Liverpool Station and compared in situ data with 3D FD analyses. They reported that prediction of lining forces using numerical modeling provides reasonable estimates; however, the actual strains tend to be more localized compared to what the numerical modeling predicts.

A chronological list of the literature reviewed is provided in Table 1. This review shows a lack of sufficient documentation regarding the magnitude of increase in hoop and longitudinal stresses within the parent tunnel lining in the vicinity of CPs. To the best of the authors’ knowledge, only limited in situ data is available, and no experimental evidence has been published on stress redistribution in tunnel linings near CPs. Furthermore, the majority of the numerical studies reviewed have been conducted under rock conditions or have modeled the surrounding strata as linearly elastic. These studies predominantly focus on overburden settlement analysis, with only a limited number addressing stress redistribution in the parent tunnel lining near CP openings. This lack of guidance leads to tunneling practitioners often relying on ground freezing or extensive structural supports around CPs to mitigate stress concentrations near the opening (Frodl 2019). Both methods, however, contribute significantly to increased costs and project delays.

**Table 1:** Chronological overview of literature related to cross-passages in tunnels

Study no	Name of study	Type of study
1	Riley (1964)	Photoelasticity
2	Pant (1971)	Photoelasticity
3	Brown & Hocking (1976)	3D BE
5	Hocking (1978)	3D BE
7	Thareja et al. (1980)	Numerical case study
8	Takino et al. (1985)	Numerical case study
9	Thareja et al. (1985)	Numerical case study
10	Gercek (1986)	Review
11	Tsuchiyama et al. (1988)	Numerical study
12	Pottler (1992)	Numerical case study
13	Swoboda et al. (1998)	Numerical case study
14	Hsiao, Y. et al. (2005)	Numerical case study
15	Sjoberg et al. (2006)	Numerical case study
16	Jones (2007)	Numerical study-field monitoring
17	Forder et al. (2008)	Case study
18	Hsiao et al. (2009)	Numerical parameteric study
19	Liu et al. (2009)	Numerical study
20	Schikora et al. (2013)	Numerical study
21	Kuyt (2014)	Numerical study-field monitoring
22	Spyridis & Bergmeister (2015)	Numerical parameteric study
23	Frodl (2019)	Case study
24	Chortis & Kavvadas (2021)	Numerical parameteric study
25	Swarup & Goel (2021)	Numerical parameteric study
26	Cohen et al. (2023)	Numerical parameteric study
27	Khetwal et al. (2023)	Numerical parameteric study
28	Siad et al. (2023a)	Numerical parameteric study
29	Siad et al. (2023b)	Numerical parameteric study
30	Thomas et al. (2023)	Numerical study-field monitoring

Therefore, this paper aims to numerically investigate the effect of cross-passage openings on the parent tunnel lining, specifically quantifying the increase in hoop and longitudinal stresses (forces-moments) around the CP opening, using three-dimensional finite element analyses. The novelty of this study lies in the use of an advance nonlinear elasto-plastic soil constitutive model to simulate the soil around the tunnel in order to quantify the increase in stresses near the CP opening and explore the effect of parameters such as the ratio of cross-passage to parent tunnel size (CP/PT), soil strength, and CP-PT intersection angle on the stress regime around the CP opening. Only results for the cover to diameter (*C/D*) ratio of 1.0 are reported since identical results are observed with a change in *C/D* ratio for shallow tunnels, as also noted by Spyridis and Bergmeister (2015). This might not hold true for deeper tunnels (*C/D* ratio greater than 2); however, the primary aim of this paper is to understand stress redistribution due to cross-passages in shallow tunnels. Conclusively, force-moment design plots are provided, which can be utilized by practitioners to modify the lining properties near cross-passages in shallow tunnels in noncohesive soils to avoid the installation of large supports or ground freezing.

The results reveal that hoop force and moment at the springlines increase significantly before CP breakout, with the highest stress arching occurring at CP/PT = 0.5. Longitudinal forces and moments follow a similar pattern, peaking around CP/PT = 0.75. At the CP crown and invert, longitudinal forces are most significant at CP/PT = 0.5, while longitudinal moments increase linearly and plateau beyond CP/PT = 0.75. Stress redistribution remains stable for intersection angles between 70 and 90°, but a 60° angle causes a notable rise in stresses, particularly at the crown and springlines. The extent of stress redistribution along the tunnel length varies with soil density, extending up to 1.5 times the CP diameter in loose soils and reducing to 1.2 times for smaller openings in denser soils.

## Methodology

**Fig. 1:**
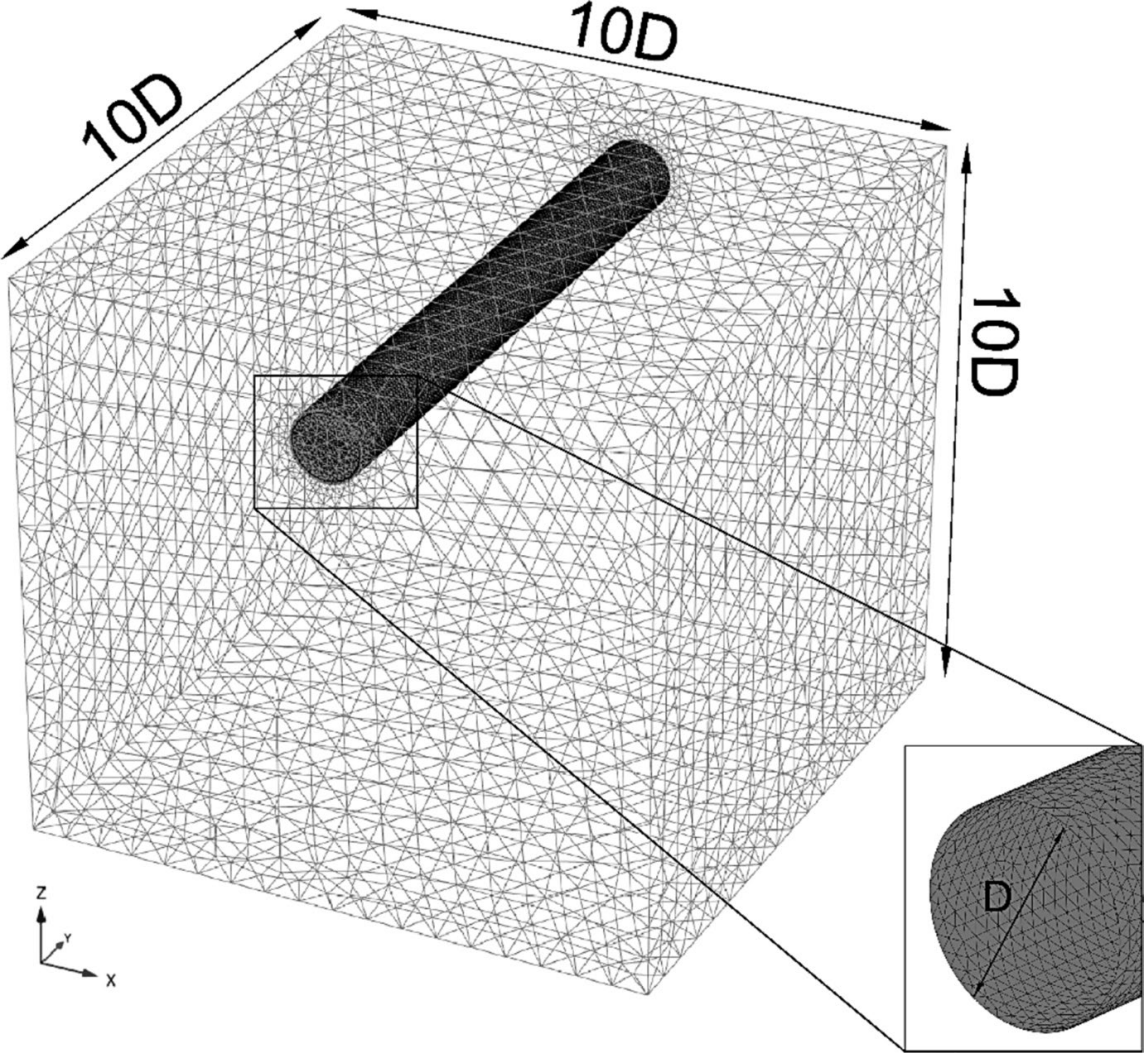
3D FE model geometry of the parent tunnel and soil mesh grid

**Fig. 2:**
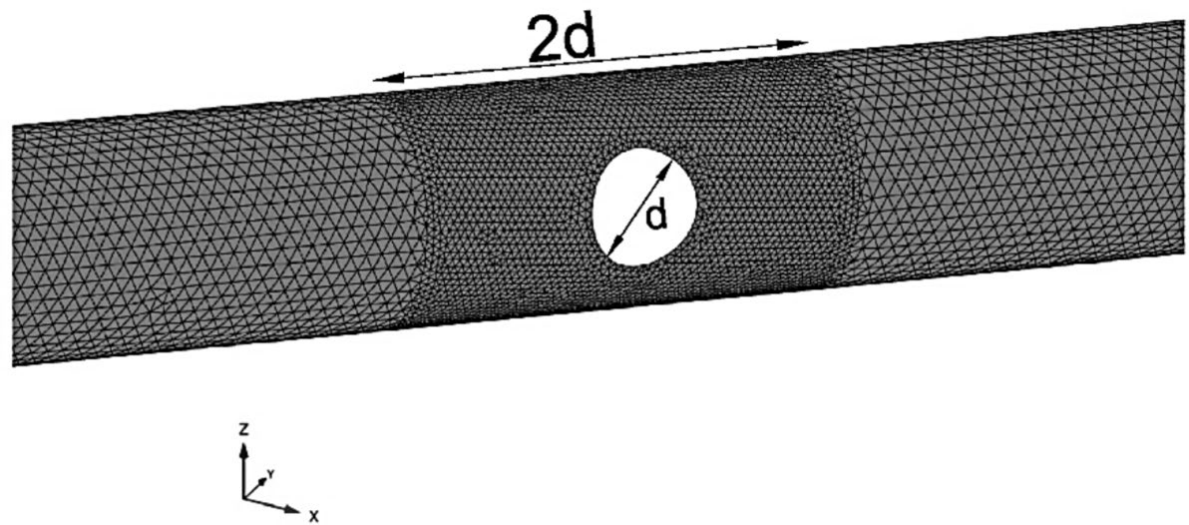
Geometry of the cross-passage and mesh discretization of the parent tunnel around the opening

A three-dimensional finite element (3D FE) approach was employed to investigate stress redistribution due to CP openings on the PT lining. PLAXIS 3D (PLAXIS, 2022; Bentley Systems (2022), a 3D FE software for analyzing geotechnical structures, was utilized for this purpose. The numerical model spanned the diameter of the parent tunnel ten times in all three directions, as depicted in Fig. 1, to minimize interference of the boundaries. The model comprised approximately 125,000 to 150,000 10-noded tetrahedral volume elements and 250,000 to 280,000 nodes, depending upon the size of the CP opening. The mesh size was refined near the CP opening (1 CP diameter longitudinally on each side), as shown in Fig. 2, so that each mesh element was equal to or smaller than the thickness of the lining (400 mm). The model was unconfined at the top, normally restrained at the sides, and fully fixed at the bottom. A total of 48 analyses were carried on varying soil strength, CP/PT ratio, and CP-PT intersection angle, as listed in Table 2, and described below.

**Table 2:** List of parameters varied in the numerical analyses

Analysis no	*D*_r_	CP/PT	Intersection angle
1	20	0.25	90°
2	20	0.50	
3	20	0.75	
4	20	0.90	
5	50	0.25	
6	50	0.50	
7	50	0.75	
8	50	0.90	
9	100	0.25	
10	100	0.50	
11	100	0.75	
12	100	0.90	
13	20	0.25	80°
14	20	0.50	
15	20	0.75	
16	20	0.90	
17	50	0.25	
18	50	0.50	
19	50	0.75	
20	50	0.90	
21	100	0.25	
22	100	0.50	
23	100	0.75	
24	100	0.90	
25	20	0.25	70°
26	20	0.50	
27	20	0.75	
28	20	0.90	
29	50	0.25	
30	50	0.50	
31	50	0.75	
32	50	0.90	
33	100	0.25	
34	100	0.50	
35	100	0.75	
36	100	0.90	
37	20	0.25	60°
38	20	0.50	
39	20	0.75	
40	20	0.90	
41	50	0.25	
42	50	0.50	
43	50	0.75	
44	50	0.90	
45	100	0.25	
46	100	0.50	
47	100	0.75	
48	100	0.90	

The parent tunnel had a diameter of 10 m and a length of 100 m, with a lining thickness of 400 mm. The CP opening is positioned at the midpoint of the parent tunnel, both cross-sectionally and longitudinally, and was modeled in four different sizes, considering CP/PT size ratios of 0.25, 0.5, 0.75, and 0.90. The following procedure is followed to analyze the numerical model and extract force-moment data:

- Initial geostatic stress state is established based on gravity.
- Excavation of soil and complete installation of parent tunnel lining (wished in place).
- Introduction of the cross-passage opening in the parent tunnel. Only the CP opening is simulated, and further soil excavation and lining installation in the cross-passage is not carried out.
- Extraction of force-moment values (hoop and longitudinal) along the cross-sections (springlines and crown). These force and moment values are normalized with the average hoop force and moment, respectively, at the springlines of the CP before breakout.
- Plotting of the largest normalized values, closest to the cross-passage against CP/PT ratio.

The analyses were conducted as “wished-in-place” simulations, where no volume loss of the PT was considered prior to the CP opening. This simplification was implemented to streamline the modeling process and save time. While ‘wished-in-place’ analyses have inherent limitations, such as inaccurate surface settlement predictions and overestimating lining loads, the former is not the focus of this research since the study is concerned primarily with the structural behavior of the tunnel lining near the CP opening. Secondly, the tendency of wished in place analysis to overestimate lining forces does lead to conservative results, as it does not account for the potential stress reduction due to volume loss. Hence, the results of this study can be considered as upper-bound estimates of lining forces for the given lining stiffness. Future studies can consider volume loss effects and lining stiffness to refine these estimates.

### Data Extraction from Numerical Models

**Fig. 3:**
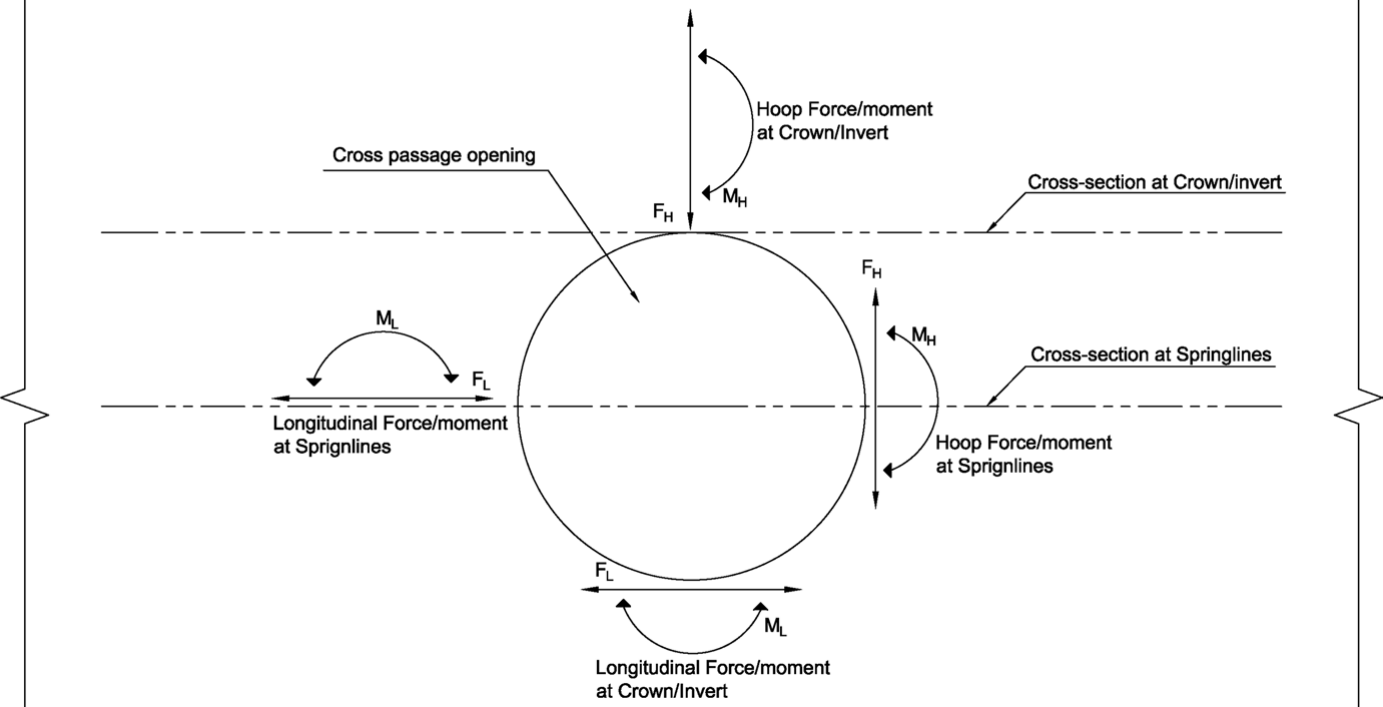
Forces and moments considered in PT lining around the CP opening

Results from 3D FE models were extracted in the form of forces and moments within the PT lining, both in the hoop and longitudinal directions, before and after the introduction of the CP opening. Two cross-sections, one at the springlines level and another at the crown height of the CP, were cut throughout the length of the parent tunnel, along which the force and moment values were extracted, as shown in Fig. 3. Since the tunnel is circular and forces within the lining at the invert are almost equal to the forces at the crown only forces at the crown are extracted.

**Fig. 4:**
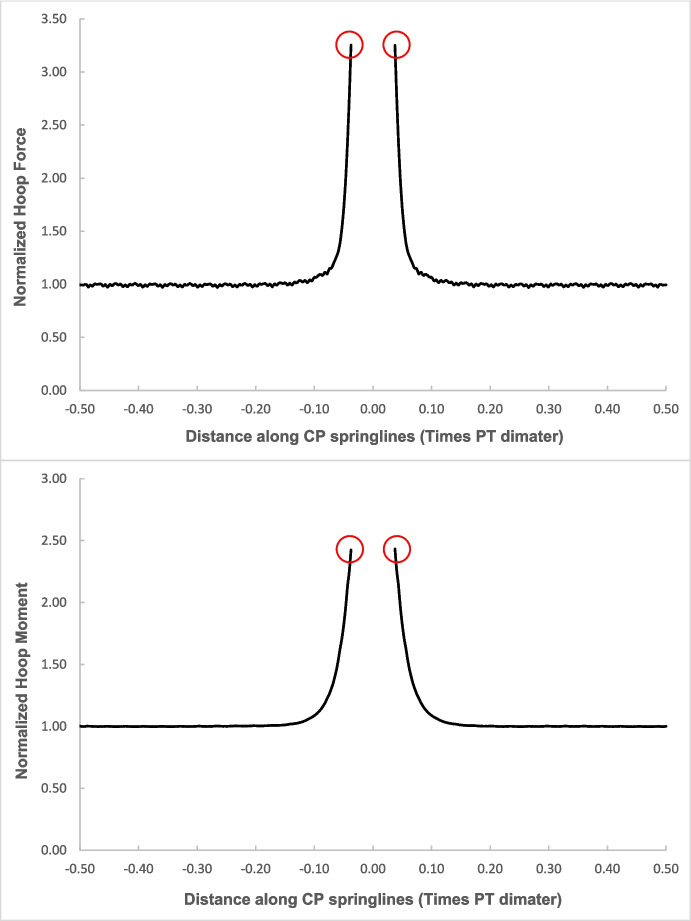
Normalized hoop forces-moments along the tunnel length at springline level of the CP (CP/PT = 0.75, Soil *E*_50_.^ref^ = 60 MPa, *C/D* = 1)

**Fig. 5:**
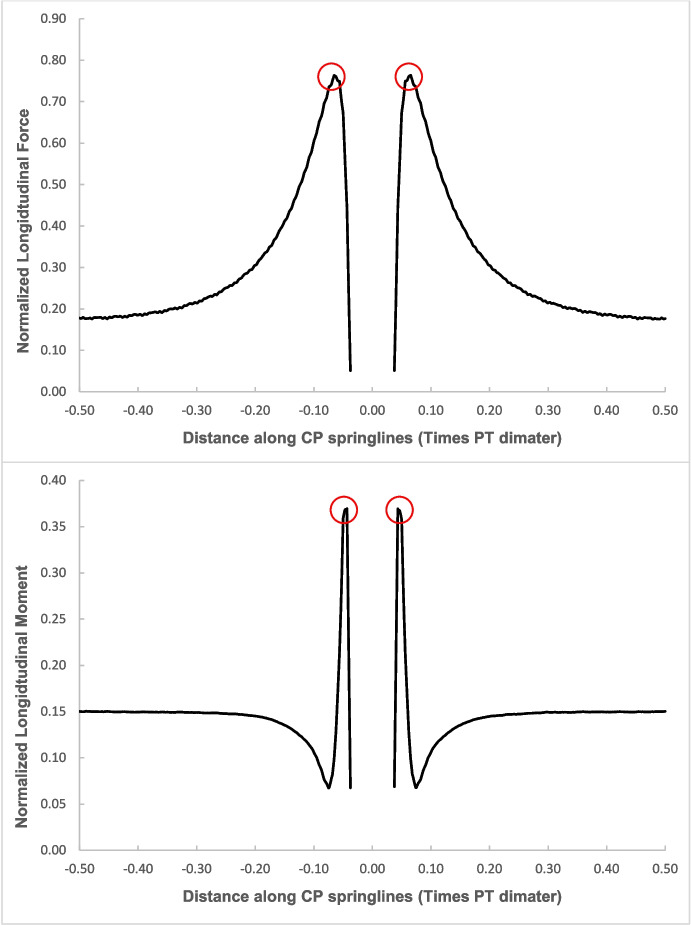
Normalized longitudinal forces-moments along the tunnel length at springline level of the CP (CP/PT = 0.75, Soil *E*_50_.^ref^ = 60 MPa, *C/D* = 1)

**Fig. 6:**
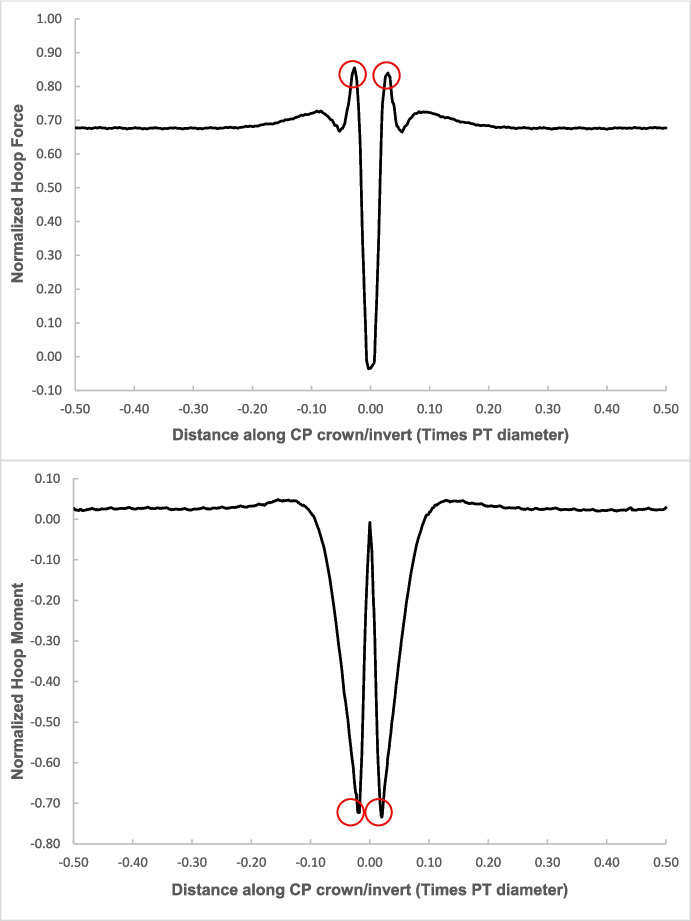
Normalized hoop forces-moments along the tunnel length at crown level of the CP (CP/PT = 0.75, Soil *E*_50_.^ref^ = 60 MPa, *C/D* = 1)

**Fig. 7:**
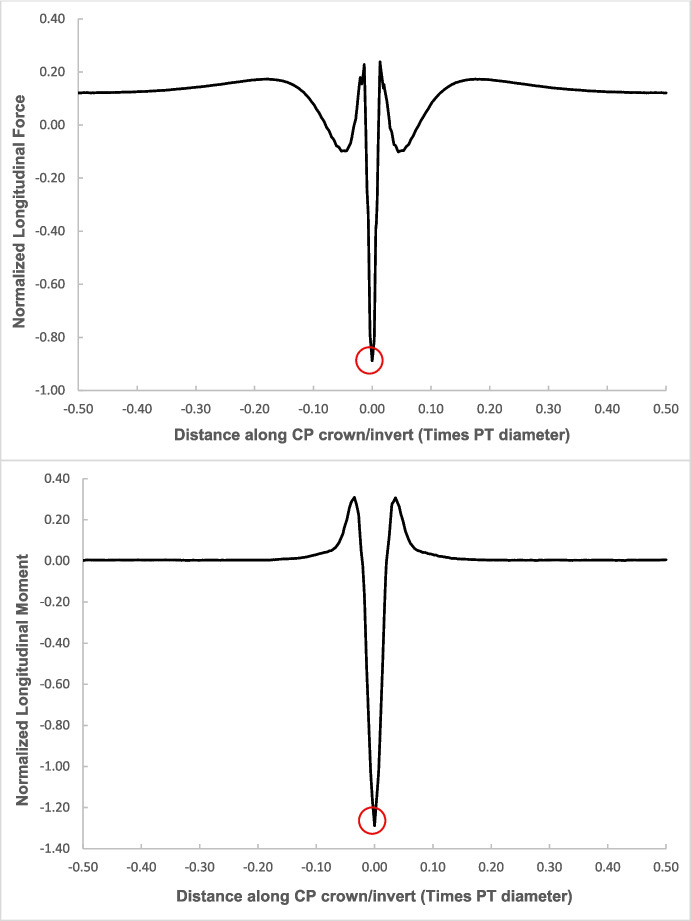
Normalized longitudinal forces-moments along the tunnel length at crown level of the CP (CP/PT = 0.75, Soil *E*_50_.^ref^ = 60 MPa, *C/D* = 1)

The force values (hoop and longitudinal) along both cross sections were then normalized with average hoop force at the springlines of PT prior to CP opening, while moment values (hoop and longitudinal) were normalized with average hoop moment at the springlines of PT before CP opening. Sample plots of these normalized force and moment distributions along both cross-sections and in both directions (hoop and longitudinal) are shown in Figs. 4, 5, 6, and 7. Finally, the largest of these normalized values, closest to the CP opening, were plotted against CP/PT ratio, as shown with the red circles in Figs. 4, 5, 6, and 7.

The reason for utilizing the average hoop force and moment at the springlines of the parent tunnel to normalize all results is that these values are easily obtainable from 2D plane-strain analyses as well. Therefore, the objective is to develop internal forces plots that can be used in conjunction with simplified 2D analysis to determine the stress regime around the CP opening. Subsequently, the derived forces and moments can be employed to design the strength, thickness, and steel reinforcement of the tunnel lining around the CP.

### Soil and Tunnel Lining Properties

The soil was modeled as a noncohesive sandy material using the nonlinear elasto-plastic constitutive model hardening soil with small strain stiffness (HSS) (Benz 2007; Vermeer 1978; Schanz et al. 1999), while the tunnel lining was modelled as a linear elastic plate element.

**Fig. 8:**
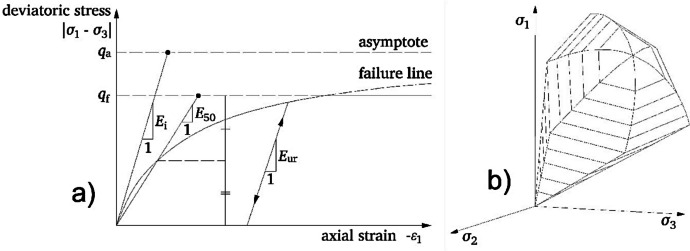
HSS model: soil response in a drained triaxial test (**a**) and yield surface in the principal stress space (**b**) (from Moller & Vermeer, 2007)

**Fig. 9:**
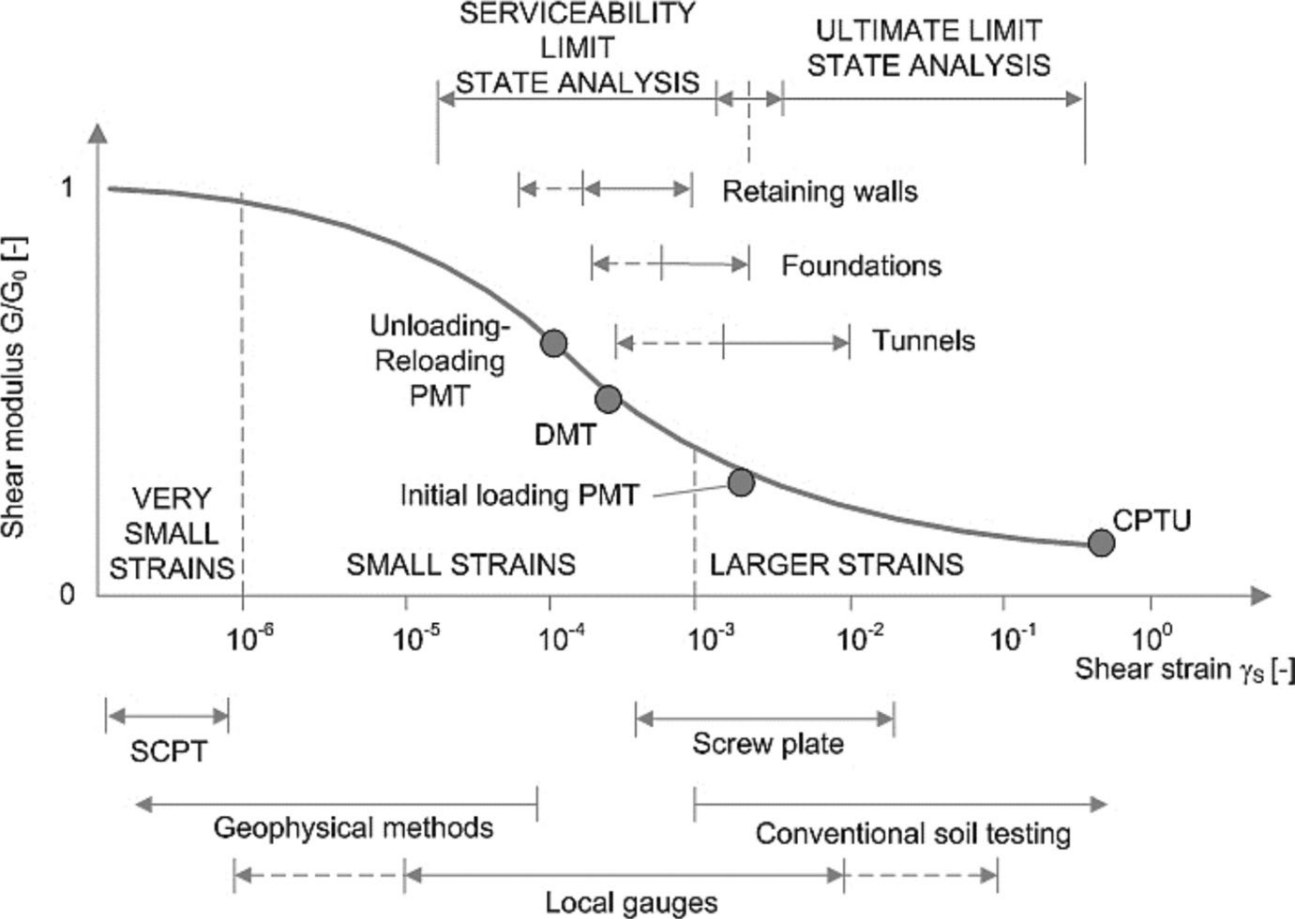
Dependence of shear stiffness on shear strain (based on Atkinson and Sallfors 1991)

The HSS is an advanced constitutive model accounting for both elastic and plastic soil behavior under varying stress conditions by incorporating stress and loading history dependent stiffness featuring both a shear and a cap yield surface. The model takes into account three different stiffness moduli (secant modulus, oedometer modulus, and unloading–reloading modulus) capturing the typical hyperbolic relation between the vertical strain ε_1_ and the deviatoric stress *q* (in a drained triaxial test) highlighted in Fig. 8a which shows a decreasing soil stiffness under loading as irreversible plastic deformations occur. The model considers hardening through both plastic shear and volumetric stress and the HS yield surface is shown in Fig. 8b. HSS also captures soil behavior even under small strains which is crucial for predicting the serviceability of geo-structures like deep excavations, foundations, and tunnels accurately as depicted in Fig. 9.

For the current study, three relative densities (*D*_r_) were considered: 20%, 50%, and 100%, representing loose, medium dense, and very dense conditions respectively. The required HSS model parameters were then determined using Eqs. 1, 2, 3, 4, 5, 6, 7,8 ,9 ,10, and 11 (Brinkgreve et al. 2010). Properties for the soil and lining are detailed in Tables 3 and 4, respectively. The modulus for the lining concrete was taken as 30 GPa.

**Table 3:** Soil properties

Parameter	*D*_r_ = 20%	*D*_r_ = 50%	*D*_r_ = 100%
*γ*_unsat_(kN/m^3^)	15.8	17	19
*γ*_sat_ (kN/m^3^)	19.32	19.8	20.6
*E*_50_^ref^ (kPa)	12,000	30,000	60,000
*E*_oed_^ref^ (kPa)	12,000	30,000	60,000
*E*_ur_^ref^ (kPa)	36,000	90,000	180,000
*G*_o_^ref^ (kPa)	73,600	94,000	128,000
*m* (-)	0.6375	0.54375	0.3875
*γ*_0.7_ (-)	0.00018	0.00015	0.0001
*ϕ*' (°)	30.5	34.25	40.5
*Ψ* (°)	0.5	4.25	10.5
*K*_0_	0.492	0.437	0.35
*R*_f_ (-)	0.975	0.9375	0.875

**Table 4:** Tunnel lining properties

Parameter	Value
Unit weight (kN/m^3^)	25
Secant modulus(GPa)	30
Poisson ratio (-)	0.15
Thickness (mm)	400

1 $${\gamma }_{\text{unsat}}\left[\frac{\text{kN}}{{\text{m}}^{3}}\right]=15+ 4.0\cdot \frac{{D}_{\text{r}}}{100}$$

2 $${\gamma }_{\text{sat}}\left[\frac{\text{kN}}{{\text{m}}^{3}}\right]=19+ 1.6\cdot \frac{{D}_{\text{r}}}{100}$$

3 $${E}_{50}^{\text{ref}}\left[\text{kPa}\right]=\text{60,000}\cdot \frac{{D}_{\text{r}}}{100}$$

4 $${E}_{\text{oed}}^{\text{ref}}\left[\text{kPa}\right]=\text{60,000}\cdot \frac{{D}_{\text{r}}}{100}$$

5 $${E}_{\text{ur}}^{\text{ref}}\left[\text{kPa}\right]=\text{180,000}\cdot \frac{{D}_{\text{r}}}{100}$$

6 $${G}_{0}^{\text{ref}}\left[\text{kPa}\right]=\text{60,000}+\text{68,000}\cdot \frac{{D}_{\text{r}}}{100}$$

7 $$m\left[-\right]=0.7- \frac{{D}_{\text{r}}}{320}$$

8 $${\gamma }_{0.7}\left[-\right]=(2- \frac{{D}_{\text{r}}}{100})\cdot {10}^{-4}$$

9 $${\varphi }{\prime}\left[^\circ \right]=28+ 12.5\cdot \frac{{D}_{\text{r}}}{100}$$

10 $$\psi \left[^\circ \right]=-2+ 12.5\cdot \frac{{D}_{\text{r}}}{100}$$

11 $${R}_{f}\left[-\right]=1- \frac{{D}_{\text{r}}}{800}$$

## Results

The 3D FE numerical model was validated by comparison with analytical closed-form solutions and 2D FE analysis before conducting the parametric study. Forces within the parent tunnel lining were assessed using the 3D model (prior to the CP opening) and compared with the 2D FE analysis and analytical models. The analytical solutions employed to evaluate the forces and moments within the tunnel lining included:

- Curtis (1976)
- Einstein and Schwartz (1979)
- Ranken, Ghaboussi, and Hendron (1978)

These analytical solutions typically assume plane strain conditions in an isotropic, homogeneous, elastic medium with an elastic lining, applicable only to circular tunnels. They also assume the lining is installed immediately (wished-in-place), which may lead to an overestimation of lining loads in real-world scenarios. However, since the 3D numerical model in this study also employs a wished-in-place circular parent tunnel with an elastic lining, it serves as a suitable validation reference. Further details related to the analytical models can be found in their respective publications. For simplicity, a C/D ratio of 2 and a relative density (*D*_r_) of 100% were used in this validation process. The soil and lining properties for the analytical solutions were consistent with those listed in Table 3.

**Fig. 10:**
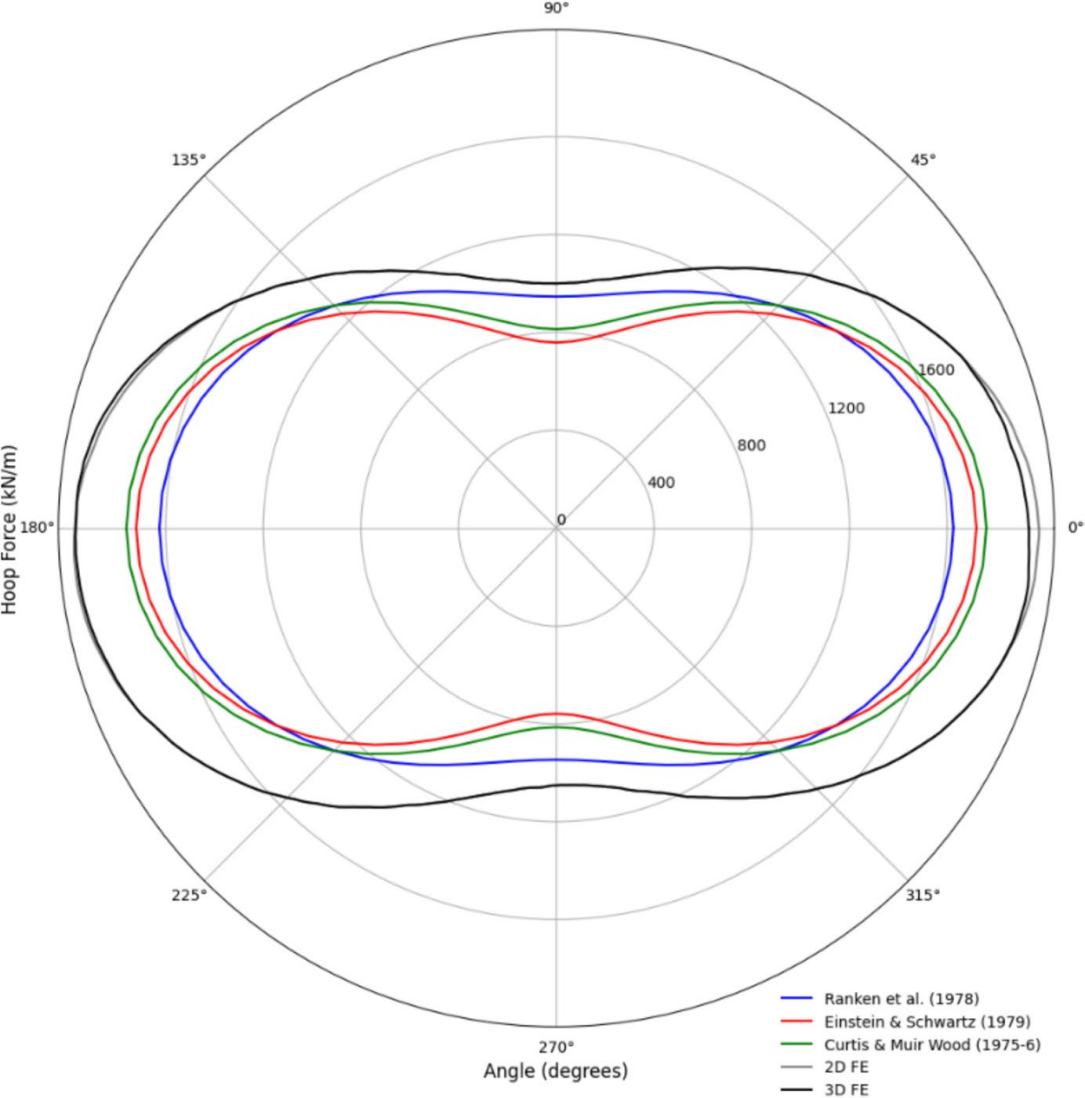
Distribution of hoop force in PT lining from analytical, 2D, and 3D results

**Fig. 11:**
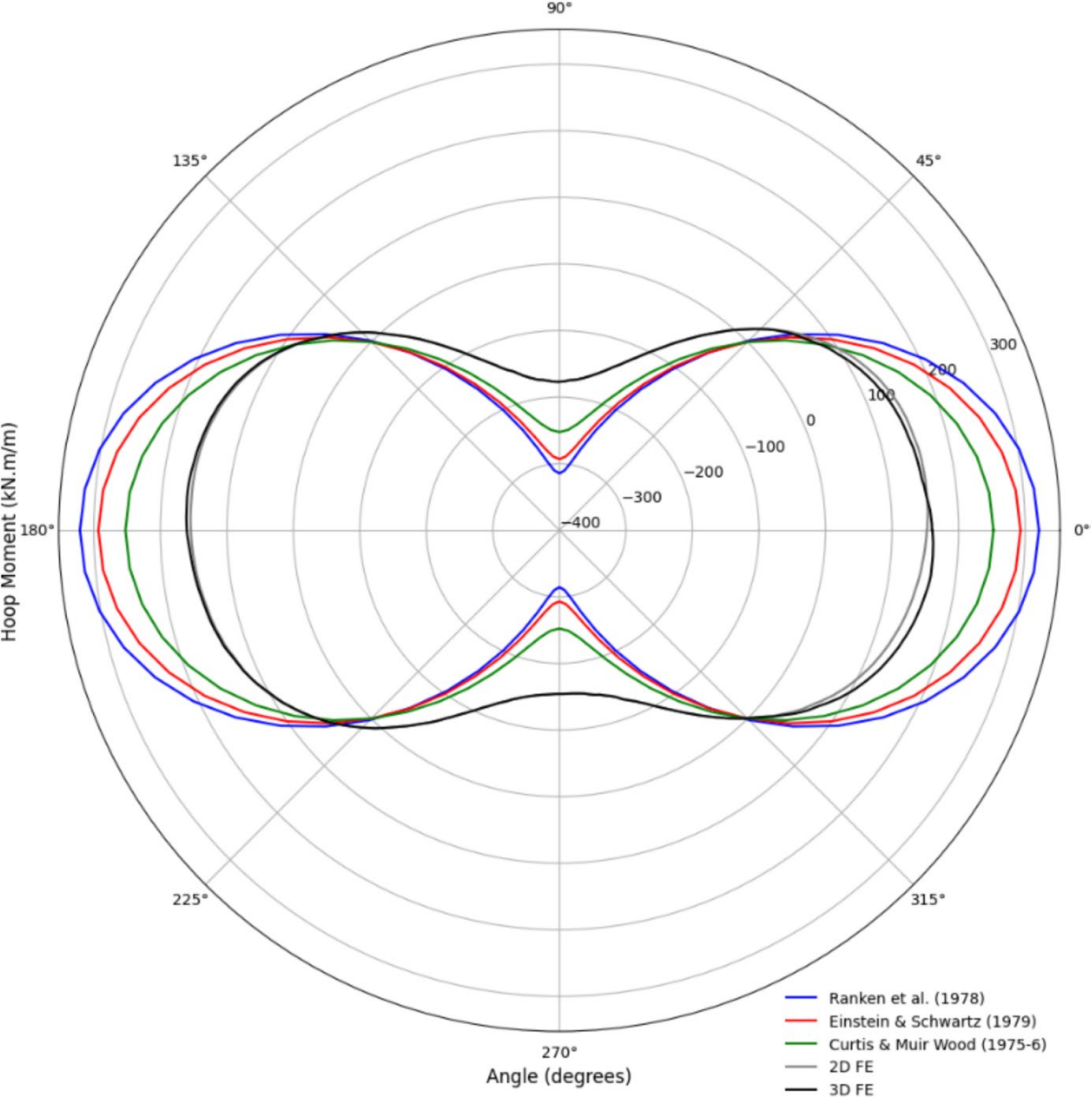
Distribution of hoop moment in PT lining from analytical, 2D, and 3D results

As shown in Figs. 10 and 11, the forces and moments within the lining derived from the analytical solutions closely match the results from the 2D and 3D analyses. The analytical solutions slightly underestimate the hoop force and overestimate the hoop moment compared to the FE analyses.

### Stress Regime at Springlines of the Cross-Passage

**Fig. 12:**
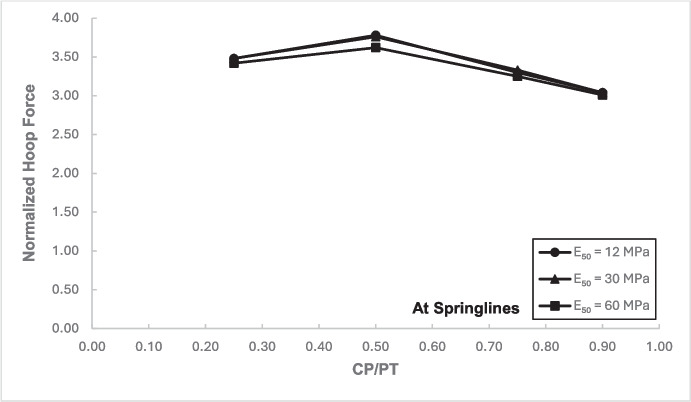
Normalized hoop force vs. CP/PT at springlines of CP

**Fig. 13:**
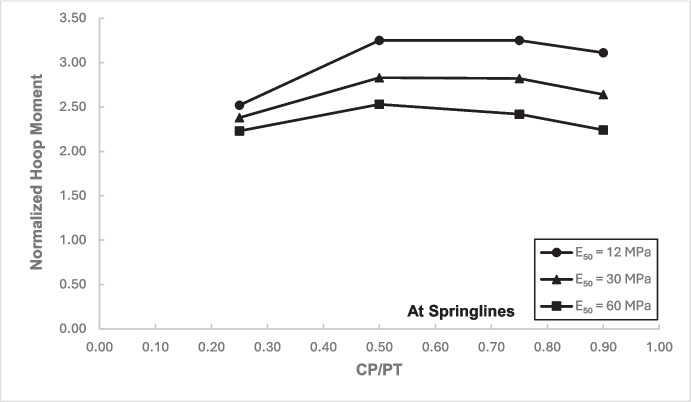
Normalized hoop moment vs. CP/PT at springlines of CP

**Fig. 14:**
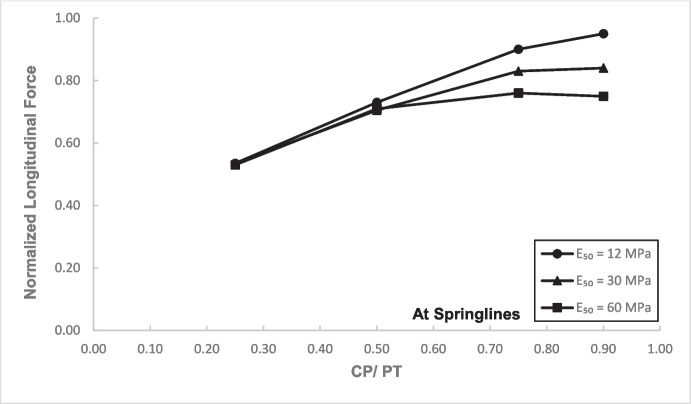
Normalized longitudinal force vs CP/PT ratio at springlines of CP

**Fig. 15:**
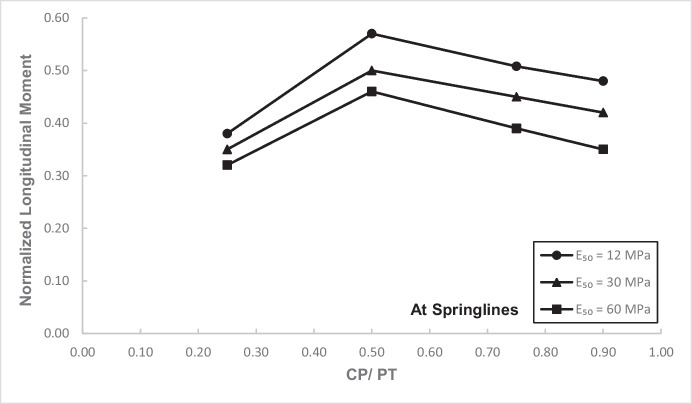
Normalized longitudinal moment vs. CP/PT ratio at springlines of CP

**Fig. 16:**
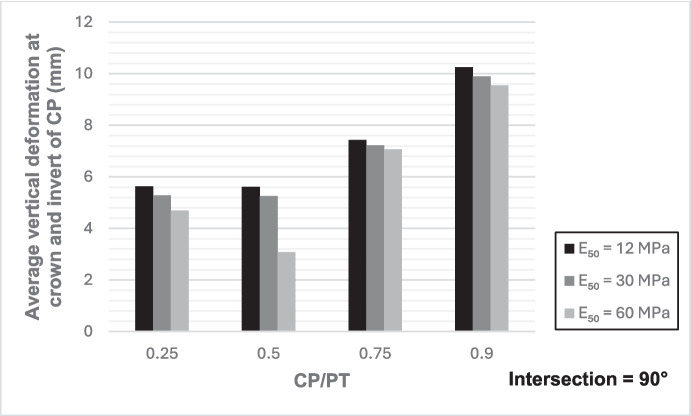
Average vertical deformation at the crown and invert of CP opening

Figures 12 and 13 show the increase in normalized hoop force and moment around the CP springlines, while Figs. 14 and 15 illustrate the change in normalized longitudinal force and moment around the CP springlines. It is evident that for any given soil strength, the maximum increase in hoop force and moment occurs if the CP/PT ratio is equal to 0.5 since a CP/PT ratio of 0.5 allows maximum arching of stresses around the CP opening. A CP/PT ratio less than 0.5 experiences a reduced stress arching around the opening as size of the opening is not large enough to allow sufficient stress redistribution. However, for openings larger than CP/PT = 0.5, the redistribution of hoop stresses is reduced due to an increase deformation at the CP crown and invert, as evident in Fig. 16. Comparing CP opening deformation with the hoop force-moment plots, it is noticeable that as the deformation increases for larger CP openings, the stress redistribution is decreased.

A similar trend of maximum value at CP/PT = 0.5 is observed for longitudinal moments, while in comparison, an almost linear increase in magnitude is observed for longitudinal forces in loose soils, while for denser soils, longitudinal forces tend to plateau after a CP/PT ratio of 0.75. Maximum longitudinal force increase is observed at a CP/PT ratio of 0.95 for all densities of soil.

For increase in soil strength, a general decrease in stresses and opening deformation is observed. Both hoop and longitudinal stresses are compressive in nature at the springlines of the CP (positive normalized values).

### Stress Regime at Crown/Invert of the Cross-Passage

**Fig. 17:**
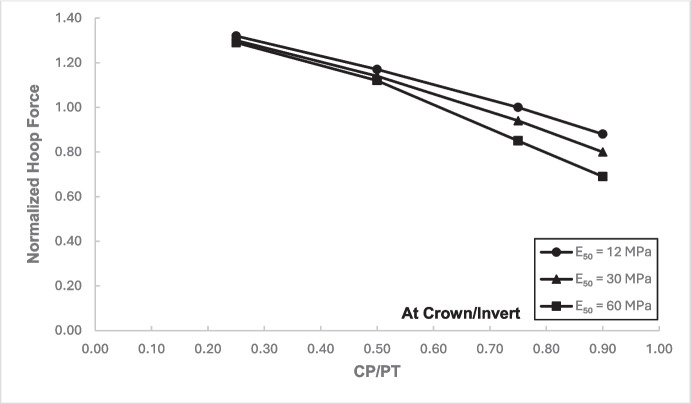
Normalized hoop force vs. CP/PT ratio at the crown/invert of CP

**Fig. 18:**
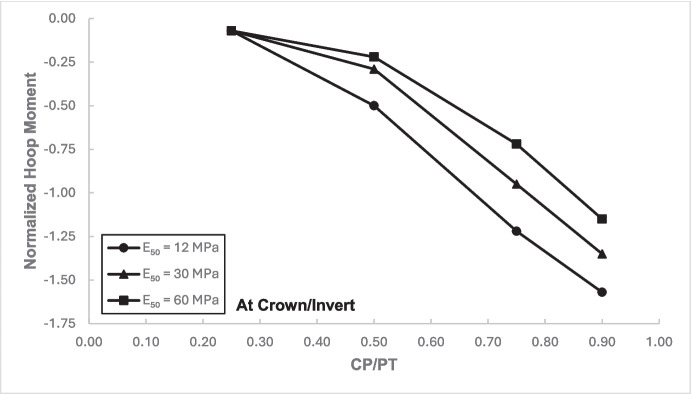
Normalized hoop moment vs. CP/PT ratio at the crown/invert of CP

**Fig. 19:**
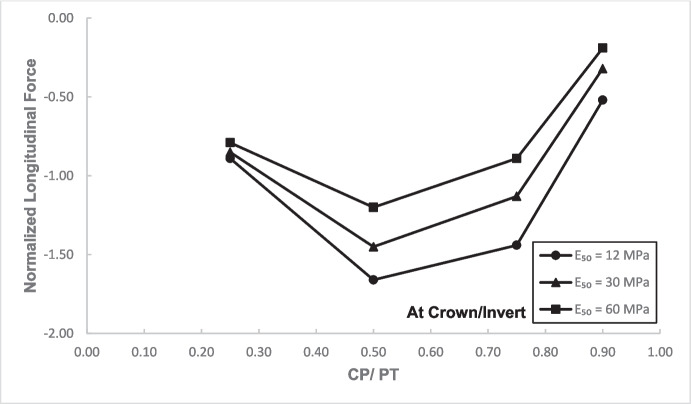
Normalized longitudinal force vs CP/PT ratio at the crown/invert of CP

**Fig. 20:**
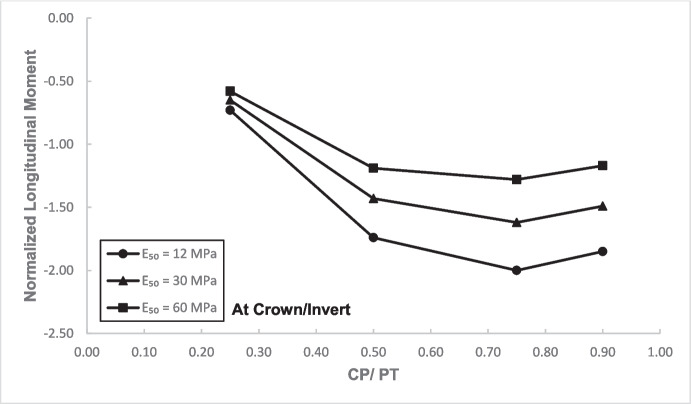
Normalized longitudinal moment vs. CP/PT ratio at the crown/invert of CP

Figures 17 and 18 illustrate the distribution of hoop forces and moments at the crown of the CP. These show a linear decrease in compressive magnitude with increasing CP/PT ratio, while hoop moments show an increase in tensile magnitude as CP/PT ratio increases. Longitudinal stresses on the other hand show that the crown experiences significant tension longitudinally (Figs. 19 and 20). The maximum tensile longitudinal force occurs at a CP/PT ratio of 0.5 while longitudinal moments increase in tension and then plateau after CP/PT ratio of 0.75.

Similar to the stress regime at the springlines of the CP, a general decrease in magnitude for hoop and longitudinal stresses is observed with increasing soil strength. Negative normalized values indicate that the crown/invert area is in tension both in hoop and longitudinal direction, except hoop force, which is compressive.

### Effect of Intersection Angle Between CP and PT

**Fig. 21:**
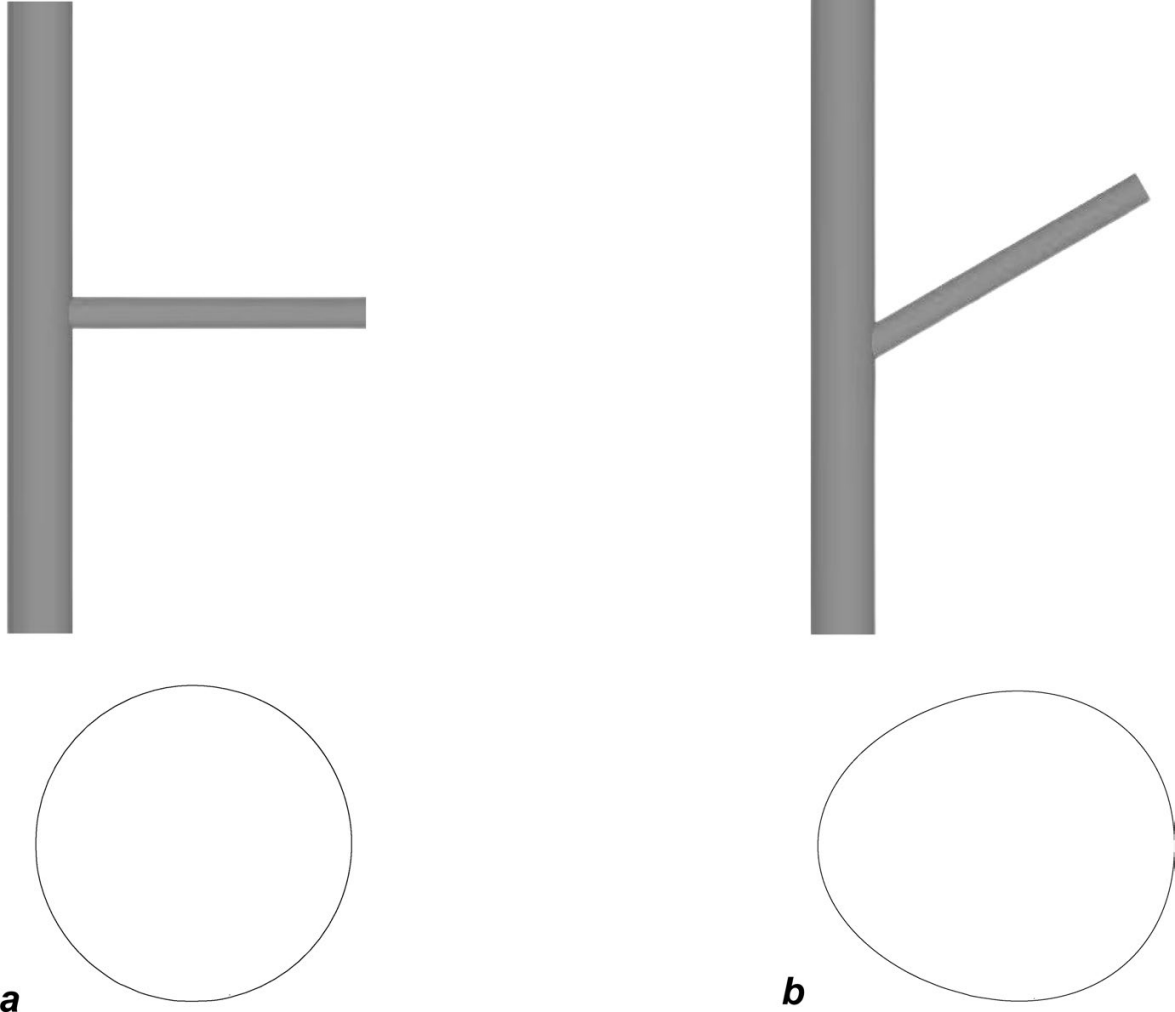
Change in shape of CP opening with change in intersection angle for intersection angle 90° (**a**) and 60° (**b**)

The intersection angle between cross-passage and parent tunnel plays an important role in the stress redistribution around CP opening as it changes the shape of the opening as shown in Fig. 21. The concave side of the opening shows a decrease in the opening area causing an increased stress concentration at this point while the convex side of the CP opening flattens and causes a decrease in stress concentration. Analyses were done for intersection angles of 60°, 70°, and 80° which were then compared to the previous results for intersection angle of 90°.

#### Stress Regime at Springlines of CP with Variable Intersection Angle

**Fig. 22:**
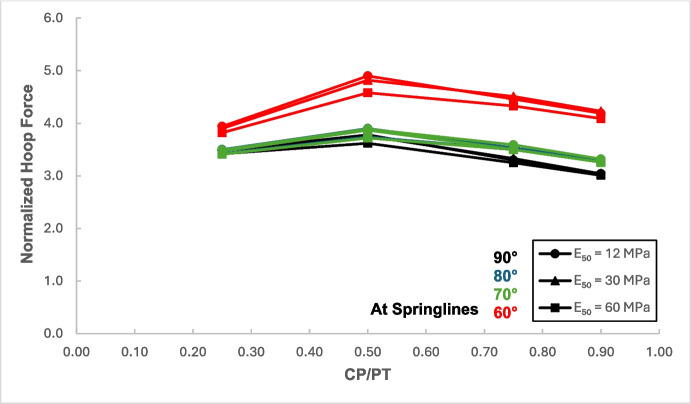
Normalized hoop force at CP springlines with varying intersection angles

**Fig. 23:**
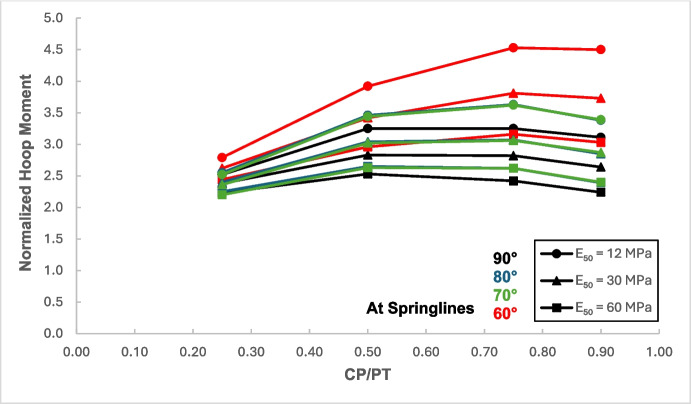
Normalized hoop moment at CP springlines with varying intersection angles

**Fig. 24:**
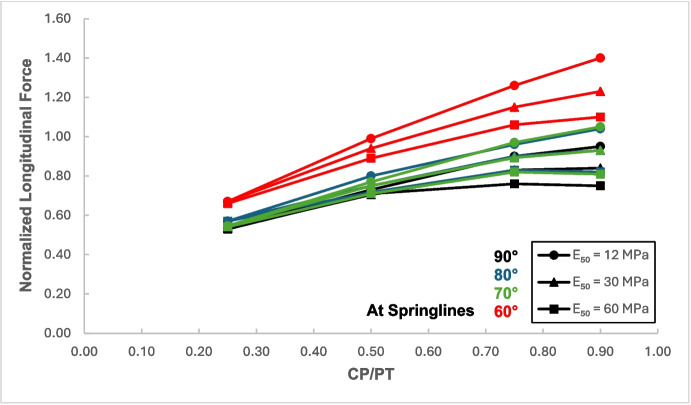
Normalized longitudinal force at CP springlines with varying intersection angles

**Fig. 25:**
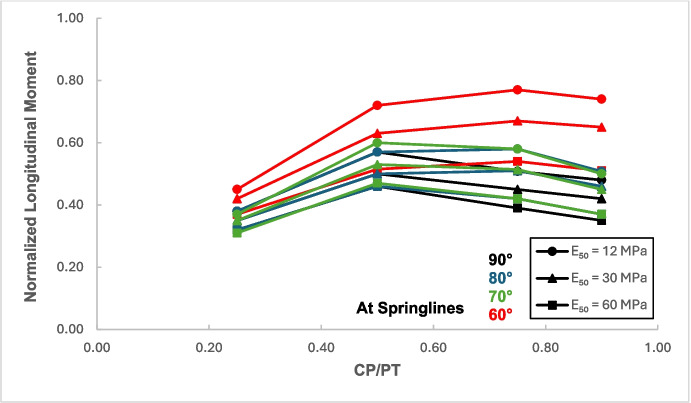
Normalized longitudinal moment at CP springlines with varying intersection angles

From the results, as depicted in Figs. 22, 23, 24, and 25, it is evident that for intersection angles of 70° and 80°, the stress redistribution at the springlines of the CP remains relatively stable and similar to that of a 90° intersection. However, for an intersection angle of 60°, a significant increase in forces and moments is observed in both the hoop and longitudinal directions.

#### Stress Regime at Crown/Invert of CP with Variable Intersection Angle

**Fig. 26:**
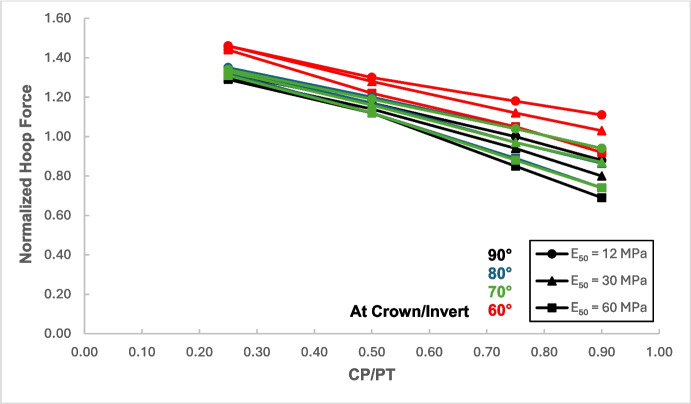
Normalized hoop force at CP crown/invert with varying intersection angles

**Fig. 27:**
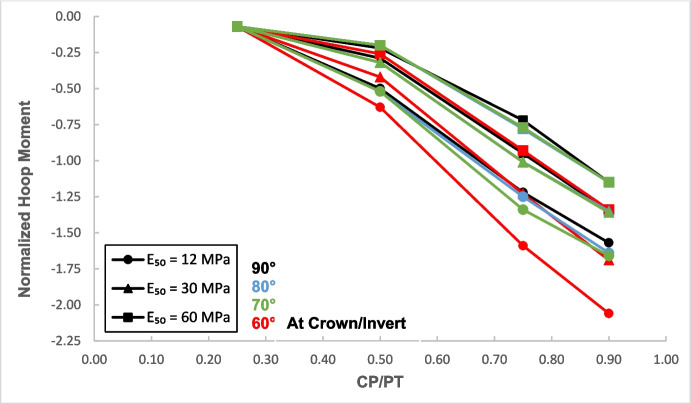
Normalized hoop moment at CP crown/invert with varying intersection angles

**Fig. 28:**
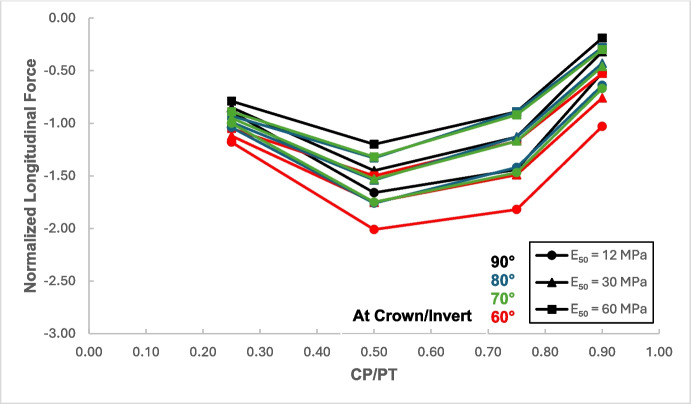
Normalized longitudinal force at CP crown/invert with varying intersection angles

**Fig. 29:**
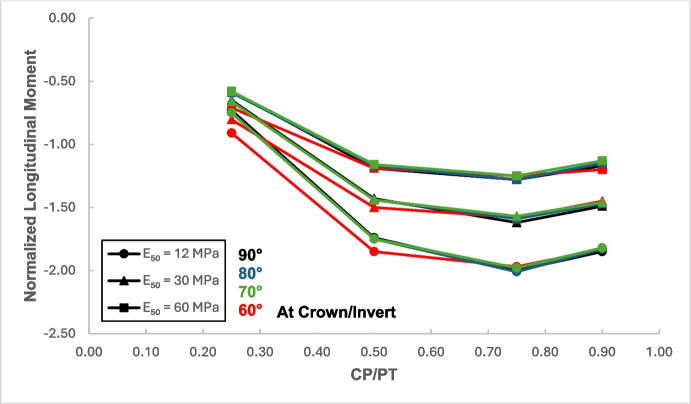
Normalized longitudinal moment at CP crown/invert with varying intersection angles

Based on the results shown in Figs. 26, 27, 28, and 29, it is apparent that hoop and longitudinal moments at the crown of CP are only minimally affected by a change in the intersection angle between CP and PT. However, there is a notable increase in the magnitude of hoop and longitudinal forces at an intersection angle of 60°. Similarly, as observed in the force-moment regime at the springlines of the CP, an intersection angle of 70° and 80° does not result in a significant increase in the magnitude of forces and moments.

### Extent of Stress Redistribution

Hereby, we refer to the region along the parent tunnel lining affected by stress increase due to the presence of the CP opening which depends on the size and shape of the CP. To determine the magnitude of this area, the analysis considered the area from the center of the CP opening to a longitudinal distance where the increase in forces and moments was reduced to only 10%. The analysis focused specifically on the redistribution of hoop forces and moments at the springlines of the PT, and an average was calculated, as these are typically the primary factors driving tunnel lining design.

**Fig. 30:**
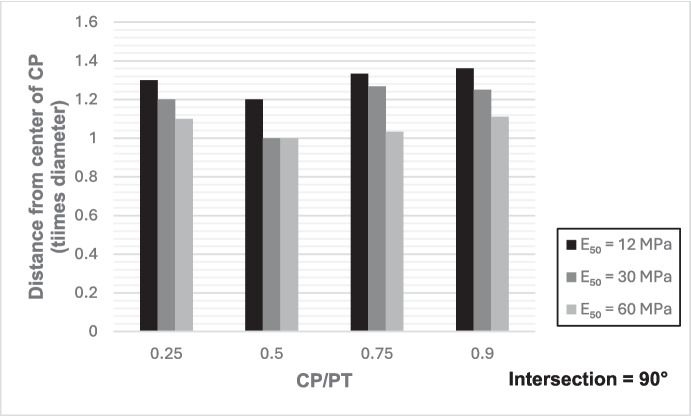
Extent of stress redistribution along the springlines for 90° intersection

**Fig. 31:**
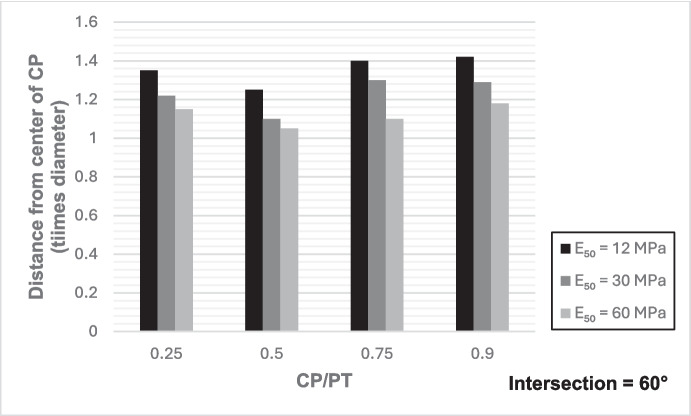
Extent of stress redistribution along the springlines for 60° intersection (concave side)

From Figs. 30 and 31, it can be observed that while the extent of stress redistribution increases with a decrease in intersection angle, the difference with a 90° intersection is only slight. However, with an increase in CP opening size, there is a general expansion in the extent of stress redistribution. A larger area of stress redistribution is noted for CP/PT = 0.25; however, this is relative to the size of the opening diameter, and a general increase in the area of stress redistribution should be anticipated with an increase in the CP/PT ratio. Additionally, an increase in soil strength leads to a decrease in the extent of stress redistribution. Nevertheless, as a rule of thumb, a maximum of 1.5 times the CP diameter for large openings in loose soils and a minimum of 1.2 times the diameter of the CP opening for smaller openings in denser soils should be anticipated.

## Discussion of Results

**Fig. 32:**
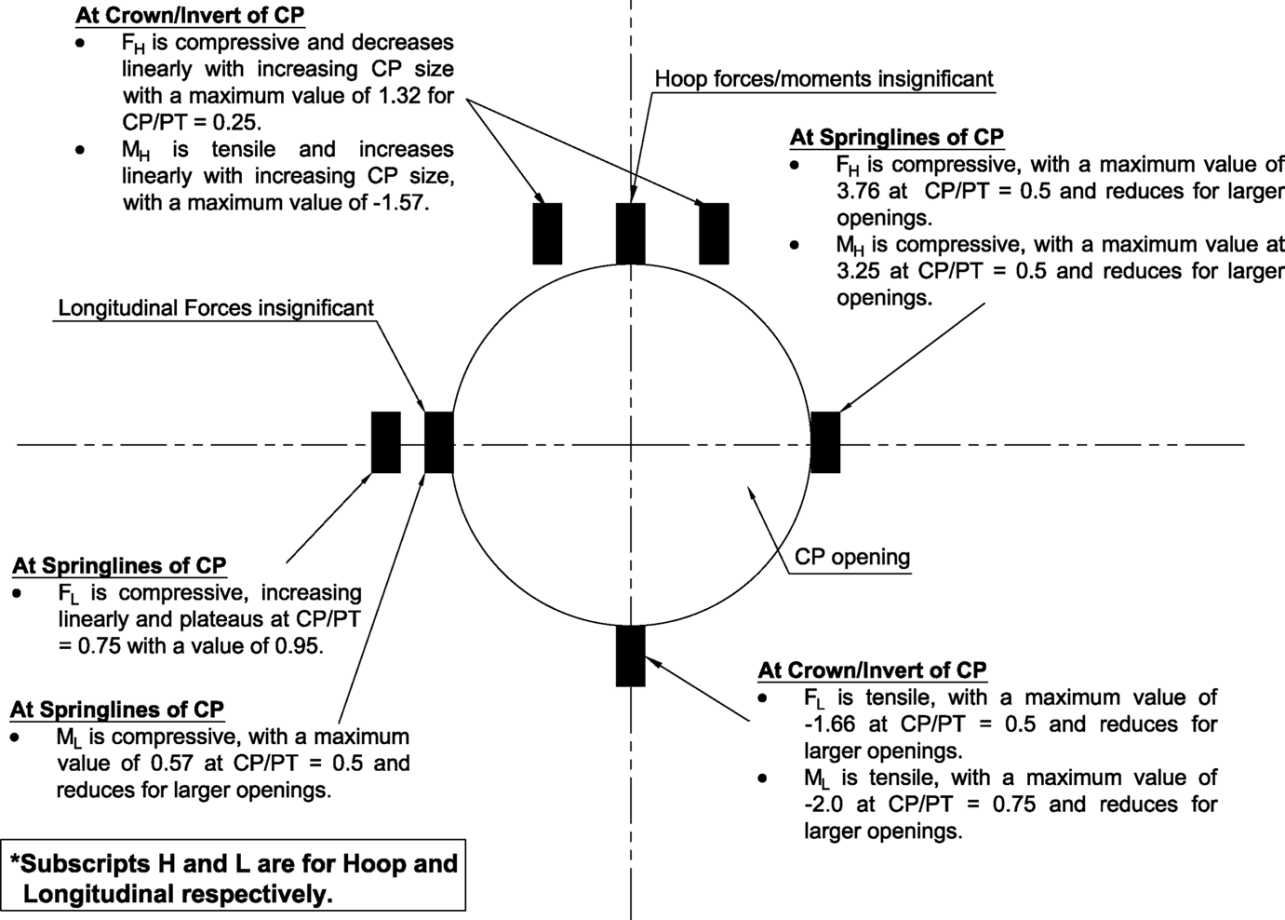
Lining forces around the CP opening when compared to hoop force and moment at springlines of PT before CP breakout

The results in Sect. 3 demonstrate the significant changes in internal forces within the parent tunnel lining following the introduction of the CP. As the CP is opened, the stress state in the parent tunnel lining is clearly altered, leading to a redistribution of internal forces. Figure 32 provides insight into the redistribution of both hoop and longitudinal forces/moments around the CP opening. The normalized force-moment (F-M) plots developed in this study (Figs. 12, 13, 14, and 15 and 17, 18, 19, and 20) can be used alongside a plane-strain 2D analysis to determine forces around a CP opening, eliminating the need for a 3D analysis. Once the internal forces around the CP are determined, the tunnel lining design can be adjusted to accommodate the increased stresses.

The force-moment plots also indicate that the CP/PT ratio has a much greater impact on stress distribution than the strength of the surrounding soil. Soil strength has a greater influence on the longitudinal force/moment at the crown and the hoop moment at the springlines of the CP. Similarly, the extent of stress redistribution is more sensitive to the CP/PT ratio than to the surrounding soil strength. Similar conclusions were reported by Spyridis and Bergmeister (2015) although they reported much smaller stress concentrations at the springlines and crown in their study. This could be attributed to the surrounding soil being modeled as an elastic medium and the *K*₀ value being assumed to be 1. Additionally, the maximum stress values were measured 350 mm (the lining thickness) away from the edge of the cross-passage, which caused the magnitude of the determined stress redistribution to be smaller.

The intersection angle does not significantly exacerbate stress concentrations around the CP unless it is less than 70°. However, as discussed in Sect. 3.3, an intersection angle of 60° increases hoop stresses by nearly 38% at the springlines and longitudinal stresses by 13.5% at the CP crown. Similar increase in stress concentrations were reported in literature (Swarup & Goel, 2021; Khetwal et al. 2023) where intersection angles of 60° and 30° caused larger settlements and increased the zone of plastic deformations compared to an intersection of 90°. This can be mitigated by maintaining the opening shape similar to that of a 90° intersection, even when the CP meets the running tunnel at an angle. However, this is primarily a construction and site issue, which is beyond the scope of this study.

## Limitations of the Current Study

The force-moment plots developed in this study are to be considered as upper bound, as the effects of volume loss and tunnel lining stiffness were not considered. Another limitation is that this study focuses on shallow conditions and assumes an at-rest pressure coefficient of less than 1. This results in vertical compression of the tunnel under loading. In contrast, with a *K*₀ greater than 1, the tunnel would experience lateral compression, altering the behavior of the lining compared to the conditions in this study. Lastly, only a circular cross-passage is modeled, and the effect of cross-passage shape is beyond the scope of this article. Future research could incorporate these factors to improve the accuracy of the current force-moment plots.

## Conclusions

This paper provides insights into the structural behavior of shallow tunnel linings, in noncohesive soils, when cross-passage openings are introduced. A 3D FE parametric study, using a nonlinear elasto-plastic constitutive model for the soil, was conducted with varying CP/PT ratio, soil strength, and CP-PT intersection angle. The analyses focused on evaluating the stress redistribution near CP openings and quantifying the increase in lining forces/moments around the CP opening.

The following conclusions can be drawn from this study:

1. The maximum increase in hoop force and moment at the springlines is found to be 3.76 and 3.25 times the average hoop force and moment, respectively, prior to CP breakout. Hoop stress arching is the largest at CP/PT = 0.5. Further increase in CP opening size reduces stress arching and increases CP opening deformation. Longitudinal forces at springlines show an almost linear increase in magnitude and tend to level off after a CP/PT ratio of 0.75. Longitudinal moments show a similar trend as hoop stresses at the springlines. Largest increase in longitudinal forces and moments is observed to be 0.95 and 0.57 times the average hoop force and moment (at springlines of PT), respectively, prior to CP breakout.
2. Longitudinal forces at the crown/invert of CP are significant and are maximum at CP/PT ratio of 0.5, while longitudinal moments increase almost linearly with increase in CP/PT ratio and plateau after CP/PT = 0.75. Largest increase in longitudinal forces and moments is observed to be − 1.66 and − 2.0 times the average hoop force and moment (at springlines of PT), respectively, prior to CP breakout. Hoop forces are compressive and decrease, while hoop moments are tensile and increase almost linearly with increase in CP size. Largest values of increase in hoop forces and moments are 1.32 and − 1.57, respectively, when compared with average hoop force and moment at the springlines of PT before CP break out.
3. Stress redistribution at the springlines of the CP remains relatively stable for intersection angles between 70 and 90°. However, for an intersection angle of 60°, a significant increase in stresses at crown/invert and springlines is observed in both the hoop and longitudinal directions. An intersection angle of 60° increases hoop stresses by nearly 38% at the springlines and longitudinal stresses by 13.5% at the CP crown.
4. The area of stress redistribution longitudinally extends to 1.5 times the CP diameter for large openings in loose soils and reaches a minimum of 1.2 times the diameter of the CP opening for smaller openings in denser soils.

## Data Availability

No datasets were generated or analysed during the current study.

## Abbreviations

CP: Cross-passage
C/D: Cover-to-diameter ratio
FE: Finite element
FD: Finite difference
PT: Parent tunnel
CP/PT: Ratio of cross-passage diameter to parent tunnel diameter
3D-BE: Three-dimensional boundary element
